# EEG 40 Hz Coherence Decreases in REM Sleep and Ketamine Model of Psychosis

**DOI:** 10.3389/fpsyt.2018.00766

**Published:** 2019-01-17

**Authors:** Santiago Castro-Zaballa, Matías Lorenzo Cavelli, Joaquin Gonzalez, Antonio Egidio Nardi, Sergio Machado, Cecilia Scorza, Pablo Torterolo

**Affiliations:** ^1^Laboratorio de Neurobiología del Sueño, Departamento de Fisiología, Facultad de Medicina, Universidad de la República, Montevideo, Uruguay; ^2^Laboratório de Pânico e Respiração, Instituto de Psiquiatria da Universidade Federal do Rio de Janeiro, Rio de Janeiro, Brazil; ^3^Laboratório de Neurociência da Atividade Física, Universidade Salgado de Oliveira, Rio de Janeiro, Brazil; ^4^The Intercontinental Neuroscience Research Group, Merida, Mexico; ^5^Departamento de Neurofarmacología Experimental, Instituto de Investigaciones Biológicas Clemente Estable, Montevideo, Uruguay

**Keywords:** gamma, schizophrenia, electroencephalogram, NMDA, cognition, dreams

## Abstract

Cognitive processes are carried out during wakefulness by means of extensive interactions between cortical and subcortical areas. In psychiatric conditions, such as psychosis, these processes are altered. Interestingly, REM sleep where most dreams occurs, shares electrophysiological, pharmacological, and neurochemical features with psychosis. Because of this fact, REM sleep is considered a natural model of psychosis. Ketamine is a non-competitive N-methyl-D-aspartate (NMDA) receptor antagonist that at sub-anesthetic dose induces psychotomimetic-like effects in humans and animals, and is employed as a pharmacological model of psychosis. Oscillations in the gamma frequency band of the electroencephalogram (EEG), mainly at about 40 Hz, have been involved in cognitive functions. Hence, the present study was conducted to analyze the EEG low gamma (30–45 Hz) band power and coherence of the cat, in natural (REM sleep) and pharmacological (sub-anesthetic doses of ketamine) models of psychosis. These results were compared with the gamma activity during alert (AW) and quiet wakefulness (QW), as well as during non-REM (NREM) sleep. Five cats were chronically prepared for polysomnographic recordings, with electrodes in different cortical areas. Basal recordings were obtained and ketamine (5, 10, and 15 mg/kg, i.m.) was administrated. Gamma activity (power and coherence) was analyzed in the abovementioned conditions. Compared to wakefulness and NREM sleep, following ketamine administration gamma coherence decreased among all cortical regions studied; the same coherence profile was observed during REM sleep. On the contrary, gamma power was relatively high under ketamine, and similar to QW and REM sleep. We conclude that functional interactions between cortical areas in the gamma frequency band decrease in both experimental models of psychosis. This uncoupling of gamma frequency activity may be involved in the cognitive features shared by dreaming and psychosis.

## Introduction

The word psychosis (from Greek: “disorder of the mind”) is used in psychiatry to define a mental state in which there is a loss of contact with reality. The “Diagnostic and Statistical Manual of Mental Disorders (DSM), 5th Edition (2013)” classifies psychotic disorders in a chapter entitled “Schizophrenia spectrum and other psychotic disorders.” Schizophrenia is the most prevalent and studied among these disorders (1). This pathology is characterized by the presence of positive symptoms (such as delusions and visual and/or auditory hallucinations, as well as disorganized behavior due to reduced ability of reality testing), negatives symptoms (apathy, loss of motivation and serious social isolation), as well as memory and executive function disorders (2–5).

Several hypotheses that attempt to explain the pathophysiology of psychotic disorders have been postulated (6, 7). One accepted hypothesis holds that glutamatergic hypofunction mediated by the N-Methyl-D-Aspartate receptor (NMDA-R) is a key mechanism contributing to positive, negative and cognitive symptoms observed in this condition (6, 8–17). This is based on clinical reports showing that the consumption of phencyclidine (PCP) or ketamine (both non-competitive antagonists of NMDA-R) induces, in healthy individuals, the characteristic alterations of the psychotic disorders, and exacerbates symptoms in patients with schizophrenia (14, 18). Therefore, the use of models involving NMDA-R hypofunction is considered a valid pharmacological model for the study of the neurobiological bases of psychotic disorders (16, 19, 20). In animals, the systemic administration of NMDA-R antagonists, such as PCP, ketamine or dizocilpine/MK-801, induce a motor behavioral syndrome, characterized by hyperlocomotion with a disorganized pattern, stereotypies, and signs of ataxia (20–22). This syndrome has been linked to the positive symptomatology of schizophrenia (12, 23). In addition, NMDA-R antagonists also produce sensory deficits and alterations in cognitive function (working and associative memory, attention) that mimic the disorders observed in psychosis; antipsychotic drugs prevent these behaviors (20–22, 24).

REM sleep is a behavioral state that has been implicated in several functions, such as brain development, learning and memory as well as in facilitating cortical plasticity. It has also been involved in increasing general creativity, and is considered critical for the well-being of the individuals (25).

Most vivid and articulate dreams occur during REM sleep (26, 27). REM sleep dreams and psychosis share important characteristics, such as internal perceptions independent of external stimulation, with a lack of criticism about the reality of the experience (28–32). REM sleep also shares neurophysiological, and neurochemical characteristics with psychosis; because of this fact, it is considered a natural model of this condition (28–30).

There are several experimental results showing that neocortical oscillations at the gamma frequency (30–100 Hz) band, mainly around 40 Hz, are involved in cognitive functions (33–35). An increase in gamma power typically appears during behaviors that are characterized by the cognitive processing of external percepts or internally generated thoughts and images (34, 36, 37). Gamma activity has been also observed during alert or attentive wakefulness (W), not only in humans, but also in animals (38–42).

The degree of electroencephalogram (EEG) coherence between two cortical regions is believed to reflect the strength of the functional interconnections that occur between them (43, 44). Recently, Cavelli et al. have proposed that the absence of EEG gamma coherence in a local activated cortical state is a conserved trait of REM sleep in mammals (45). In fact, although gamma power is relatively high, gamma coherence is lost during REM sleep in cats, rats and humans (46–49). Interestingly, gamma coherence values during lucid dreaming are between wakefulness and REM sleep (47), and self-awareness in dreams can be induced through frontal low current stimulation at gamma frequency (50).

The similarities of the cognitive functions during REM sleep and under the effect of ketamine, highlights the importance of comparing the EEG gamma activity in both experimental models of psychosis. Hence, the aim of the present study is to compare gamma (30–45 Hz) power and coherence that reflect short and long-range gamma synchronization, respectively (45, 46), during REM sleep with the effect induced by subanesthetic doses of ketamine.

## Materials and Methods

Five adult cats were used in this study; four of them were also utilized in a previous report (51). The animals were obtained from and determined to be in good health by the Institutional Animal Care Facility. All experimental procedures were conducted in accord with the *Guide for the Care and Use of Laboratory Animals* (8th edition, National Academy Press, Washington DC, 2011) and were approved by Institutional and National Animal Care Commissions (Protocol N° 070153-000089-17). Adequate measures were taken to minimize pain, discomfort or stress of the animals. In addition, all efforts were made to use the minimum number of animals necessary to produce reliable scientific data.

**Figure 1:**
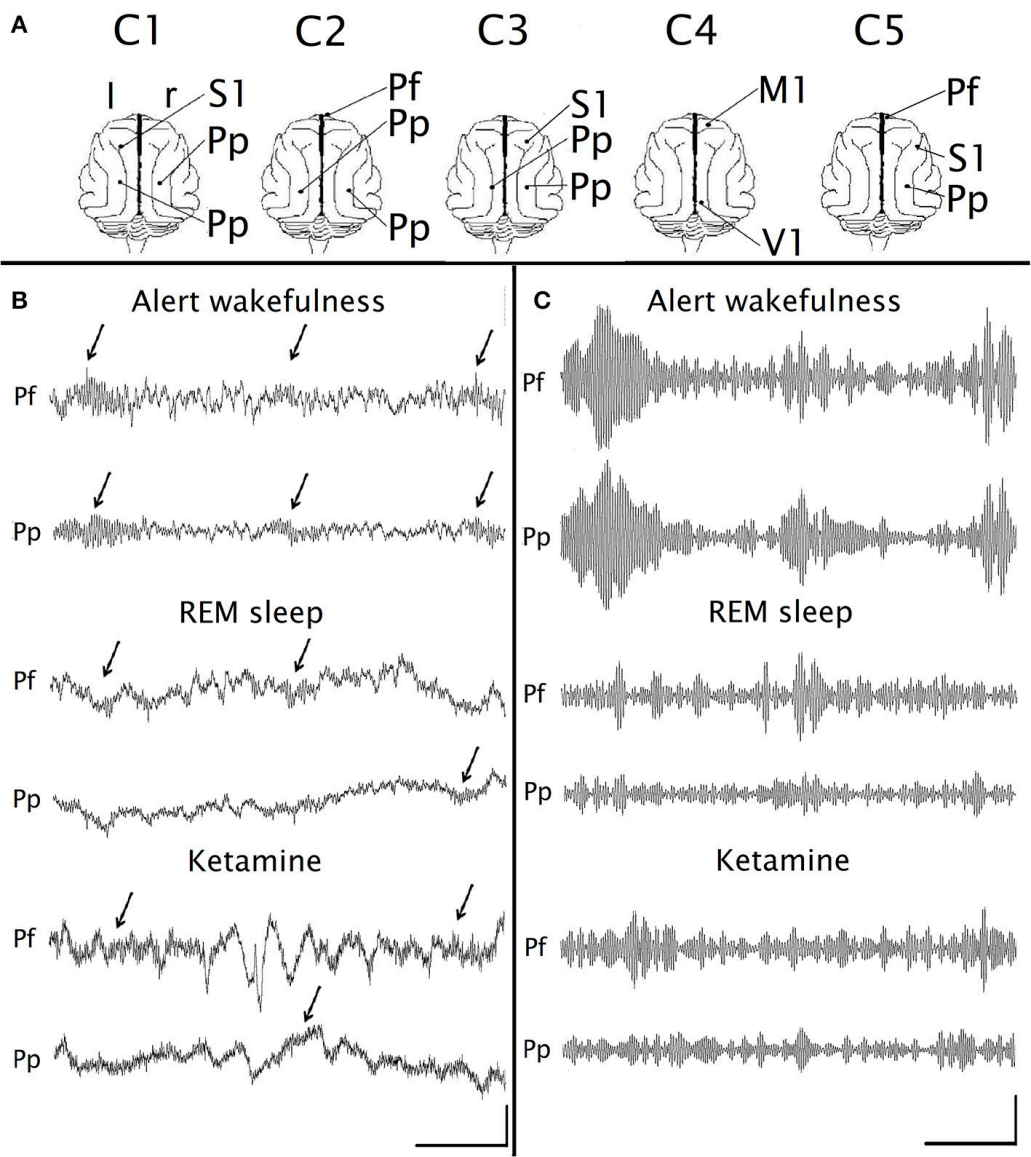
Gamma EEG oscillations under the effect of ketamine. **(A)** Position of the recording electrodes on the surface of the cerebral cortex. The recordings were referenced to an indifferent common inactive electrode located on the frontal sinus. C1-C5, identification name of the animals. M1, primary motor cortex; Pf, prefrontal cortex; Pp, posterior-parietal cortex; S1, primary somato-sensory cortex; V1, primary visual cortex; r, right; l, left. **(B)** Simultaneous raw recordings from a representative animal of the prefrontal (Pf) and parietal posterior (Pp) cortices during alert wakefulness, REM sleep and under the administration of ketamine (15 mg/kg). The arrows indicate the gamma “bursts.” **(C)** Filtered recordings (bandpass 30–45 Hz) of the same recordings as in **(B)**. Calibration bars: 1 s, 200 μV for **(B)**; 1 s, 20 μV for **(C)**.

The surgical procedures were the same as in previous studies (48, 51, 52); hence, only a brief description of the surgical procedures will be done. Following general anesthesia, the head was positioned in a stereotaxic frame and the skull was exposed. Stainless steel screw electrodes (1.4 mm diameter) were placed on the surface (above the dura matter) of different cortical areas. Figure 1A shows the sites of the recording electrodes used in this study. Note that because the animals were not prepared specifically for this work, we did not analyze exactly the same cortices in all of them. The electrodes were connected to a Winchester plug, which together with two plastic tubes were bonded to the skull with acrylic cement in order to maintain the animals' head in fixed position without pain or pressure. After recovery from surgical procedures, they were adapted to the recording environment for a period of at least 2 weeks.

Experimental sessions of 4 h were conducted between 11 a.m. and 3 p.m. in a temperature-controlled environment (21–23°C). During these sessions (as well as during the adaptation sessions), the animals' head was held in a stereotaxic position by four steel bars that were placed into the chronically implanted plastic tubes, while the body rested in a sleeping bag (semi-restricted condition).

The EEG activity was recorded with a monopolar (referential) configuration, utilizing a common reference electrode located in the left frontal sinus. The electromyogram (EMG) of the nuchal muscles, which was recorded by means of acutely placed bipolar electrode, was also monitored. The electrocardiogram (ECG), by electrodes acutely placed on the skin over the pre-cordial region, and respiratory activity by means of a micro-effort piezo crystal infant sensor were also recorded. Each cat was recorded daily for ~30 days in order to obtain complete basal and treatment data sets.

Bioelectric signals were amplified (× 1,000), filtered (0.1–500 Hz), sampled (1,024 Hz, 2^16^ bits) and stored in a PC using the Spike 2 software (Cambridge Electronic Design).

Data were obtained after ketamine administration as well as during spontaneously occurring quiet wakefulness (QW), non-REM (NREM) sleep and REM sleep. Alert wakefulness (AW) was induced for a period of 300 s by sound stimuli; in drug-free recordings, the stimuli were introduced ~30 min after the beginning of the recording sessions. The sound consisted of clicks (0.1 ms in duration) of 60–100 dB SPL with a variable frequency of presentation (1–500 Hz, modified at random) in order to avoid habituation (48, 53). In occasions, AW was induced by visual stimulation, by means of placing a mirror in front of the animal for 300 s (48). It is important to note that in one animal (C4), the sleep and W data was the same as the one used in Torterolo et al. (51). In other three animals, data were obtained from recordings performed specifically for the present study, but from animals that were also utilized in Torterolo et al. (51). The fifth animal was not used in previous studies.

Five, 10, and 15 mg/kg i.m. of ketamine (Ketonal^Ⓡ^, Richmond Veterinaria S.A.) were administered to five animals. These three doses were administered four times in each animal in different experimental sessions in a counterbalanced order (each animal received 16 doses of ketamine). The recovery time between consecutive ketamine experiments was 72 h.

Ketamine (50 mg/ml) was diluted in benzethonium chloride, hydrochloric acid, and water (solution for veterinary use). In pilot experiments, saline was injected (to rule out that the effect obtained was due to the pain caused by the injection) and the EEG analysis showed similar results to those observed in baseline recordings; therefore, basal recordings (without injections) were used as control.

Sound stimuli during 300 s were applied 10 min after ketamine injection (Supplementary Figure 1). These sound stimuli had the same characteristics as those used to induce AW (48).

**Figure 2:**
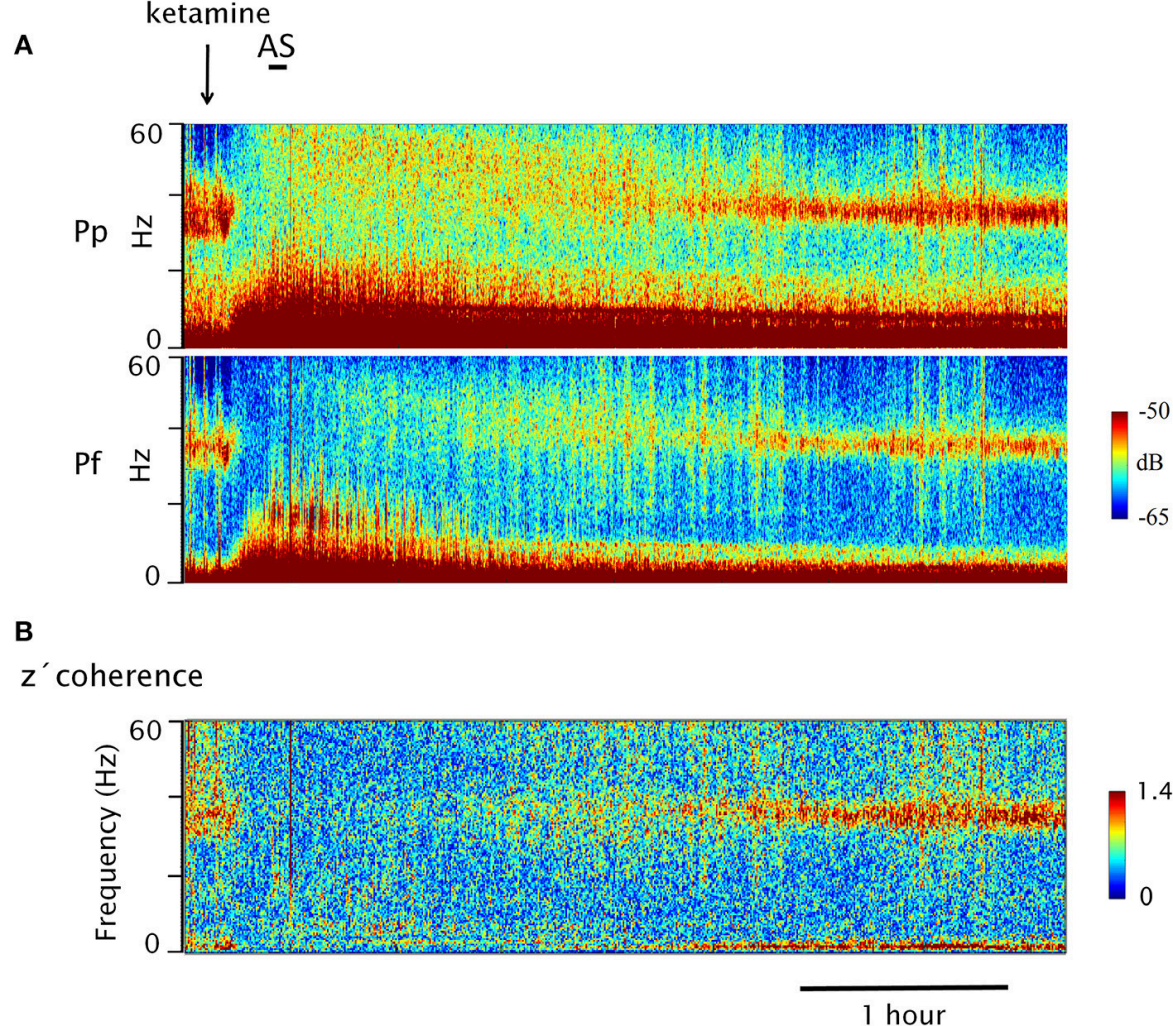
Dynamic evolution of EEG gamma power and z′ coherence following the injection of ketamine. **(A)** Gamma power spectrograms of prefrontal (Pf) and posterior parietal (Pp) cortices following ketamine injection (arrow) in a representative animal (C5). The horizontal bar represents sound stimulation (AS). Power is represented in a color code. **(B)** Coherogram of the gamma z′-coherence between Pf and Pp cortices (same recordings as in **A**). Time and the frequency are displayed on the horizontal and vertical axes (depth), respectively; the z-coherence is represented in a color code.

**Figure 3:**
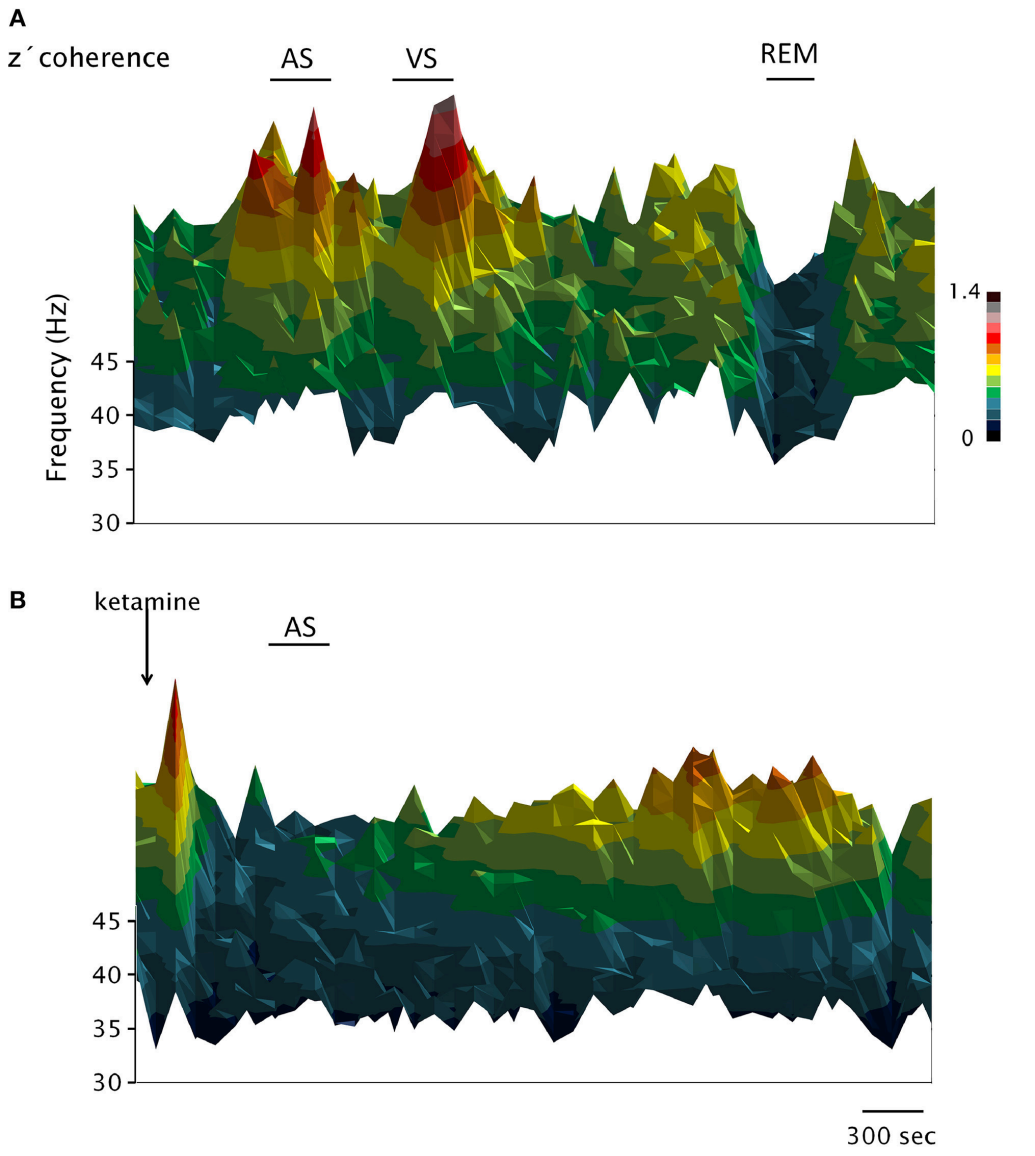
Dynamic evolution of the EEG gamma z′-coherence during wakefulness, sleep and under the effect of ketamine. 3D coherograms of the gamma z′-coherence of simultaneous recordings of the prefrontal and posterior-parietal cortices of C2. The horizontal axis indicates the time, the vertical (depth) axis indicates frequency while the z′-coherence is represented by color code. **(A,B)** Represent the coherograms of a basal recording and following the administration of ketamine (15 mg/kg), respectively. AS, sound stimulus; VS, visual stimulus.

Data were analyzed as in our previous studies (48, 51, 52). Sleep and W were quantified in epochs of 10 s. In order to analyze coherence between pairs of EEG electrodes, 12 artifact-free periods of 100 s were examined during each behavioral state (1,200 s for each behavioral state). For each pair of recordings, data were obtained during four recording sessions following ketamine administration, and from four basal (without injections) recording sessions for AW, QW, NREM, and REM sleep. Gamma activity following ketamine administration was evaluated in windows taken during the stimulus, and temporarily moved away 300 s from it (see Supplementary Figure 1). These windows were chosen during the maximum effect of ketamine (between 5 and 20 min after the injection, see Figures 2, 3).

For each selected period of 100 s, Magnitude Squared Coherence was analyzed for the low gamma frequency band (30–45 Hz) by means of Spike 2 script COHER 1S (Cambridge Electronic Design) (see 48, for details in the definition of coherence). This period of analysis was divided into 100 time-blocks with a sample rate of 1,024 Hz, a bin size of 2,048 samples and a resolution of 0.5 Hz. We employed the same time-windows as for the coherence analysis, to process gamma power (by means of the Spike 2 script COHER 1S). Recordings were also filtered (band pass 30–45 Hz) using Spike 2 digital finite impulse response filters. Averages of the signals were also performed.

**Figure 4:**
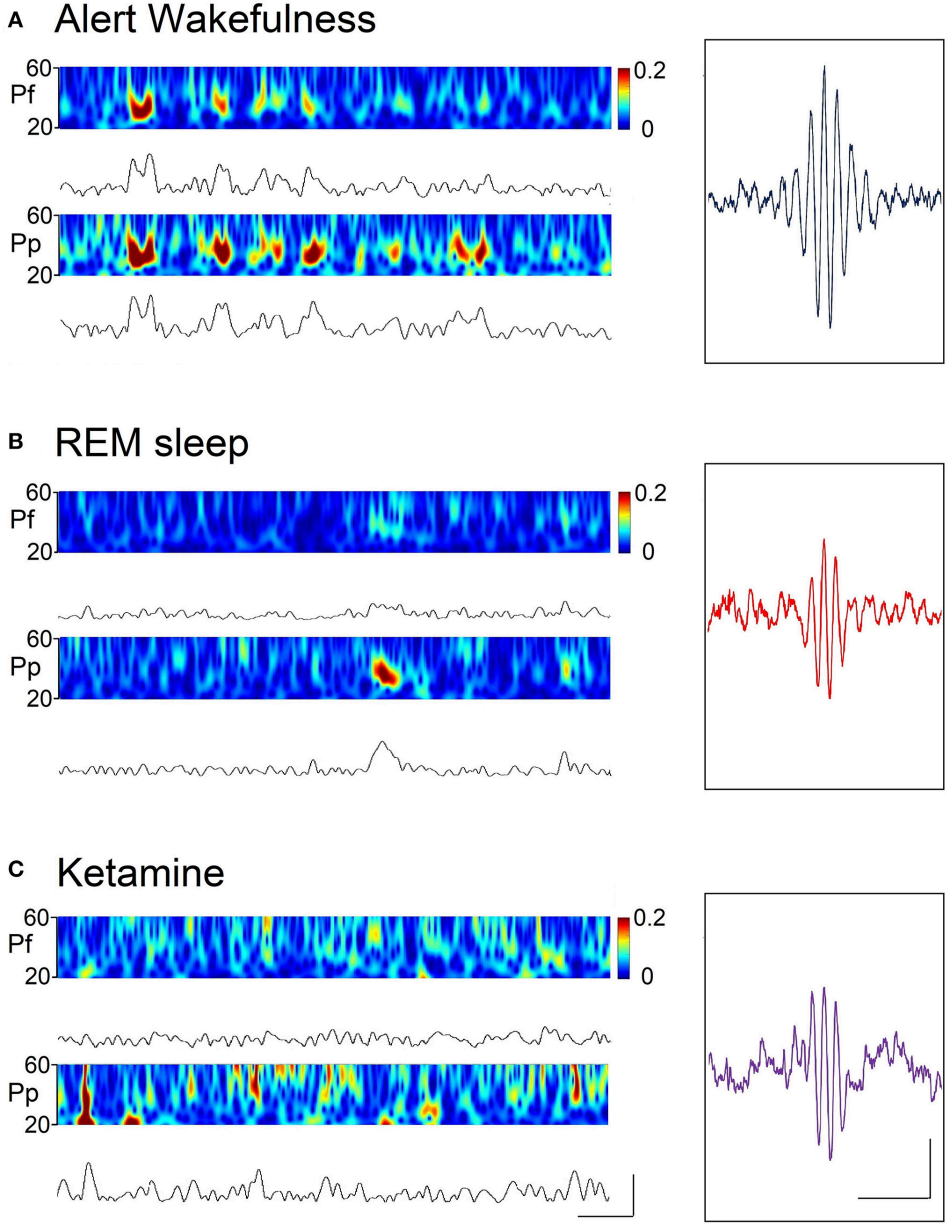
Spectrograms (by means of the wavelet function) and rectified gamma (30–45 Hz) band or envelopes, during alert wakefulness **(A)**, REM sleep **(B)**, and following the administration of ketamine (15 mg/kg) **(C)** of a representative animal. Calibration bars; 400 ms and 30 μV. Insets. Gamma “bursts” averaged from a filtered recording (high-pass 3 Hz) selected from the prefrontal cortex. Hundred random bursts were selected and averaged; the “trigger” was the peak of the wave with greater amplitude of the “burst.” Calibration bars: 200 ms and 10 μV.

In order to improve the quality of the Figures, in Figures 2, 4 two different approaches were used in order to display the EEG power. In Figure 2 a multitaper method was used as described by Babadi and Brown (54). This method utilized a series of discrete prolate spheroidal sequences (Slepian) for the Fast Fourier Transform. The procedure reduces the variance of the power spectrum estimate, offering a better power estimation. On Figure 4, a wavelet transformed was applied in order to improve time and frequency localization. Morlet wavelet was utilized because of its proven suitability for EEG analysis (55). Both of these analyses were performed employing the Chronux Toolbox and the ND Tools Toolbox, running on self-built MATLAB routines.

The mean power and z′-coherence of the gamma band in each EEG channel or derivative (pair of EEG channels) were calculated for every behavioral state and drug treatments. The significance of the differences among conditions was evaluated for each cat with one-way ANOVA and Tamhane *post-hoc* test. In order to analyze the gamma coherence averaged in the whole group of animals, the mean intra-hemispheric z′-coherence of the gamma band between anterior (S1 for C1 and C3, Pf for C2 and C5, and M1 for C4; see Figure 1A) and posterior (V1 for C4, and Pp for the rest of the animals) cortices was evaluated. For this purpose, we utilized the repeated measures ANOVA (rmANOVA) and Bonferroni *post-hoc* test. The criterion used to reject null hypotheses was *p* < 0.05.

## Results

Following the administration of the different doses of ketamine, the animals were awake with their eyes wide open; hence the doses employed were below anesthesia threshold. However, especially with higher doses, after 5 min they stopped tracking the experimenters with their eyes. When the animals were put back in freely moving condition (at the end of the experiments, 3–4 h after the injection of ketamine), they were able to ambulate.

The results will be described in the following way. First, the different effects of the highest dose (15 mg/kg) will be presented. Thereafter, a dose-response curve will be shown.

### Raw and Filtered Recordings

Figures 1B,C show raw and filtered (band pass 30–45 Hz) simultaneous recordings of the prefrontal and posterior parietal cortices, during AW, REM sleep and following ketamine administration (15 mg/kg). In AW, “bursts” of gamma oscillations appear simultaneously in both channels (arrows in Figure 1B), while during REM sleep these oscillations have lower amplitude and duration, and do not occur simultaneously in both channels. Under the effect of ketamine administration, gamma oscillations have very similar characteristics to those observed during REM sleep. As was described before (56, 57), some slow waves were also present after the injection of ketamine (Figure 1B); the analysis of these waves was out of the scope of the present study.

## Dynamic of the Gamma Power and Coherence Following Ketamine Administration

In Figure 2, the power and coherence analyses of a representative recording of C5 are shown after ketamine administration (15 mg/kg). Figure 2A shows the dynamic evolution of the power. First, there was a brief period of high gamma activity in the narrow 30–45 band triggered by the injection pinch; thereafter, the gamma power was first reduced and then increased to encompass a much wider range of frequencies (between 30 and 60 Hz). After 2 h, large values of 30–45 Hz power begins to reappear. Figure 2B exhibits the dynamic evolution of the z′-coherence between the prefrontal and parietal-posterior cortices. Following a brief increase in gamma coherence (post-injection alertness), it decreased to very low levels. The sound stimulus (labeled as AS in Figure 2A) had no effect, neither on the power nor on the gamma coherence. Approximately 2 h following the administration of the drug, gamma coherence began to increase, and reach values similar to AW about 3 h after the injection.

Figure 3 shows the dynamic evolution of the z′-coherence between prefrontal and posterior-parietal cortices in two representative recordings of C2 (basal recording in Figure 3A, and following the administration of ketamine in Figure 3B). In the recording shown in Figure 3A, sound (AS) and visual (VS) stimulation were performed. After that, the animal had periods of sleep (an episode of REM sleep is indicated). Both the AS and VS caused an increase in gamma coherence; during REM sleep the coherence decreases to a minimum level. In Figure 3B, the effect of ketamine (15 mg/kg) is shown. After a brief increase in gamma coherence due to the injection pinch, it decreased down to REM sleep level. The sound stimulation also had no effect on gamma coherence. The changes in gamma power and coherence induced by ketamine displayed in Figures 2, 3 were observed in all the animals; C5 and C2 had the longest and shortest duration of the effect, respectively.

In order to analyze more deeply the characteristics of the gamma activity during AW, REM sleep and under the effect of ketamine, gamma envelopes and spectrograms are displayed in Figure 4. In this Figure, it is visually simple to compare the dynamics of the “gamma bursts” among conditions. A marked coupling can be observed between the cortices during AW, whereas this coupling disappears both during REM sleep and under ketamine. Also, the average waveforms of the gamma bursts during REM sleep and under the effect of ketamine are similar (Figure 4, insets); these “bursts” are different from those of AW.

### Mean Gamma Power and Coherence

Under ketamine, when contrasting the values of power and gamma coherence during the windows affected by the sound stimulus and not affected by it, there were no significant differences (Figures 2, 3; quantitative data not shown). Therefore, to simplify, only the analysis of the windows not affected by the sound stimulus will be described.

**Figure 5:**
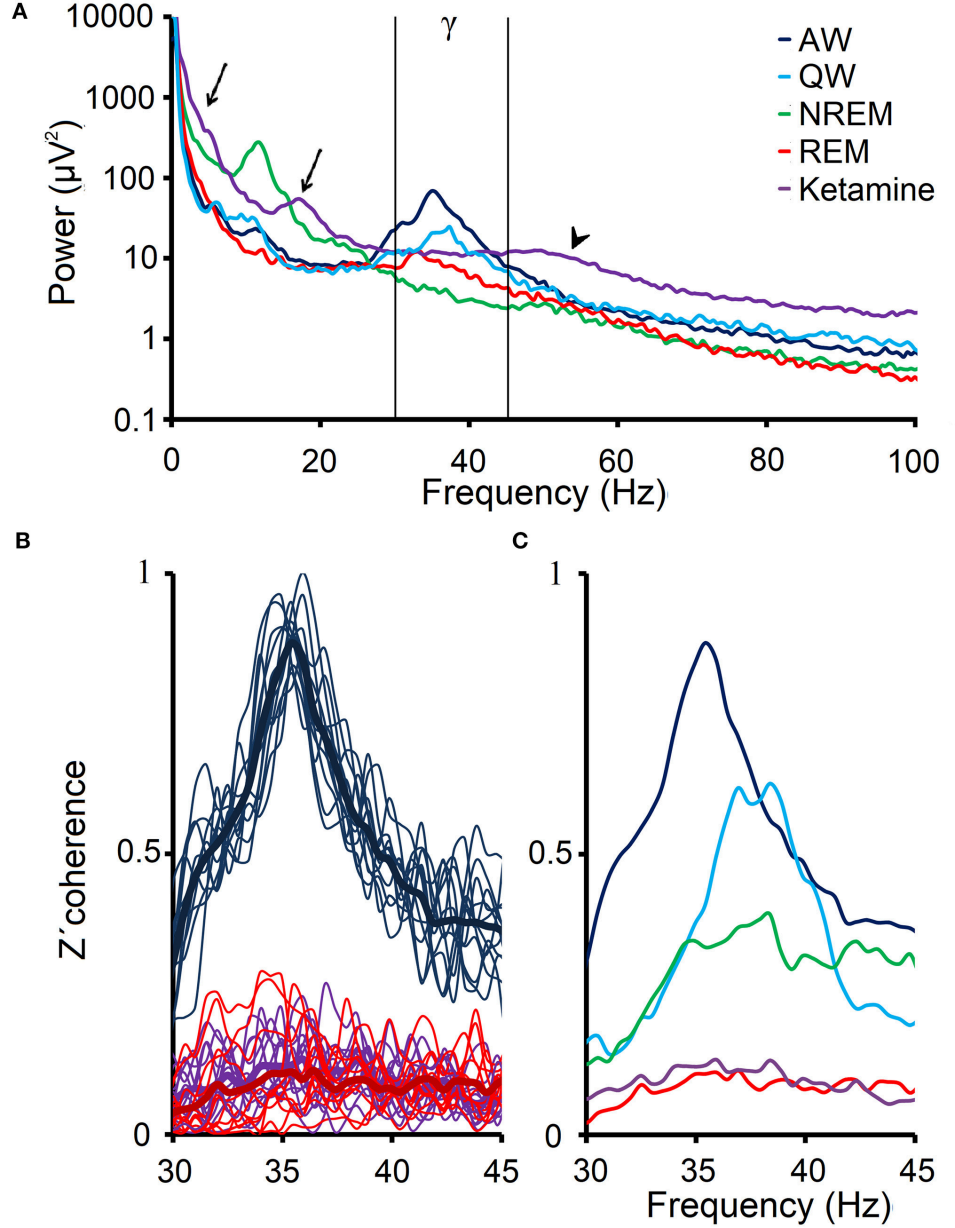
EEG gamma power and coherence under the effect of ketamine. **(A)** Power spectrum (0.5–100 Hz) that was obtained from the average of ten 100 s windows, from the prefrontal cortex EEG of C5 during alert (AW), quiet wakefulness (QW), NREM and REM sleep as well as following the administration of ketamine (15 mg/kg). The arrows show the delta and beta power peaks under ketamine, and the arrowhead shows the high gamma power under the drug. Low gamma band is shown between vertical lines. **(B)** 12 profiles of the z′-coherence (thin lines) between the prefrontal and posterior-parietal cortical areas of the C5, as well as the averages (thick lines) of these 12 profiles for AW, REM sleep and following administration of ketamine. **(C)** Average gamma z′-coherence profiles for the same conditions as in **(A)**.

Figure 5A shows the analysis of the power spectrum of the prefrontal cortex of C5 during W, sleep and under ketamine. Ketamine generates alterations in the low frequency bands and a small peak in the beta band (arrows). Focusing on gamma band, we observed that 30–45 Hz gamma band power under ketamine is lower than AW, but comparable to QW or REM sleep. However, the gamma power at frequencies above 45 Hz (high gamma, arrowhead) is greater than in physiological states.

Gamma power values for each cat and cortex are shown in Table 1. The power under ketamine (15 mg/kg) are comparable to QW and REM sleep; in most cases, are minor than AW and larger than NREM sleep.

**Table 1:** Gamma (35–40 Hz) power values during sleep, and wakefulness and 10 min following ketamine administration.

**Animal/Cx**		**AW**	**QW**	**NREM**	**REM**	***K***	***F***
**C1**	**Pp**	64.4 ± 2.4	18.3 ± 4.7	8.9 ± 0.3	15.1 ± 0.5	28.0 ± 0.7^abcd^	66
	**S1**	15.3 ± 0.6	13.1 ± 0.9	7.6 ± 0.3	13.0 ± 0.6	6.8 ± 0.1^abd^	7
**C2**	**Pf**	60.9 ± 3.1	23.8 ± 3.6	12.5 ± 0.3	20.9 ± 0.4	14.5 ± 2.6^abd^	50
	**Pp**	57.6 ± 2.6	21.1 ± 3.9	10.3 ± 0.3	18.5 ± 0.5	24.7 ± 1.4^ac^	37
**C3**	**Pp**	14.0 ± 0.8	10.8 ± 0.9	6.5 ± 0.6	9.7 ± 1.3	10.7 ± 0.3^ac^	26
	**S1**	26.2 ± 1.5	13.1 ± 0.9	6.1 ± 0.4	7.7 ± 0.5	15.4 ± 0.5^acd^	131
**C4**	**M1**	75.8 ± 4.3	26.7 ± 0.9	15.8 ± 0.5	19.6 ± 0.9	25.5 ± 0.9^ac^	73
	**V1**	57.4 ± 3.0	8.4 ± 0.6	5.8 ± 0.91	10.2 ± 0.5	9.8 ± 0.2	55
**C5**	**Pf**	132.0 ± 4.0	57.2 ± 5	20.5 ± 1.3	18.3 ± 1.1	33.4 ± 1.8^abcd^	113
	**S1**	121.2 ± 4.9	69.8 ± 4.4	18.6 ± 1.8	37.0 ± 2.4	45.6 ± 2.8^abcd^	121
	**Pp**	174.0 ± 11.2	79.8 ± 4.4	35.3 ± 3.2	39.9 ± 2.5	64.8 ± 3.4^acd^	85

*The values represent mean ± standard error. The letters a, b, c, and d show the significance (p < 0.05, ANOVA followed by Tamhane tests) compared to ketamine (K); a vs. AW, b vs. QW, c vs. NREM, and d vs. REM sleep. All the analyses have the same degrees of freedom (4 between groups, 55 within groups). Right cortices are shown for C2–C5, while left cortices are exhibited for C1. C1–C5 identification name of the animals. M1, primary motor cortex; Pf, prefrontal cortex; Pp, posterior-parietal cortex; S1, somatosensory cortex; V1, primary visual cortex; Cx, cortex*.

Figure 5B illustrates the z′-coherence profiles between prefrontal and posterior- parietal cortices of C5 during AW, REM sleep and under the effect of ketamine (15 mg/kg). The z′-coherence profiles under ketamine were similar to those during REM sleep. Figure 5C shows the average coherence profiles of the gamma band for all physiological and pharmacological states in this representative animal (C5). Gamma z′-coherence under the effect of ketamine was as low as during REM sleep; gamma z′-coherence in these conditions was lower compared to the others behavioral states.

**Figure 6:**
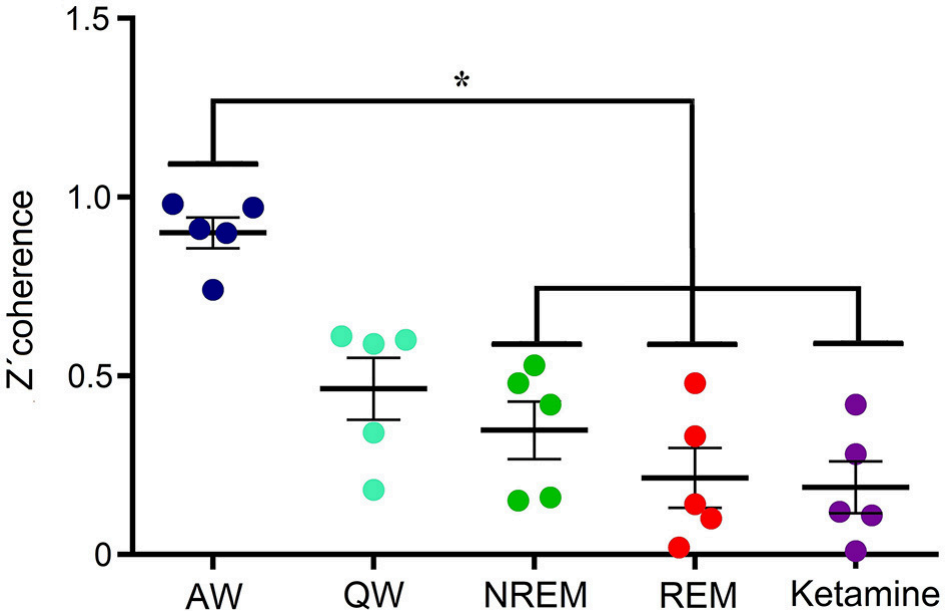
Gamma z′-coherence between anterior and posterior cortical areas for the five animals during alert (AW) and quiet (QW) wakefulness, NREM and REM sleep, as well as following 15 mg/kg of ketamine. Error bars indicate standard error. *F_(4, 24)_ = 75.4, *p* < 0.001.

Figure 6 shows, for all animals, the gamma coherence between the anterior and posterior cortices for all behavioral states and ketamine. The level of z′-coherence under ketamine was similar to REM sleep. Although the rmANOVA only distinguished between ketamine and AW, when we analyzed each animal individually there were significant differences between all the states. Table 2 displays the z′-coherence for all the combinations of cortices and for all the animals. Ketamine decreases gamma coherence down to REM sleep levels (or even lower) for all the combinations of cortices studied of the five animals.

**Table 2:** Gamma (35–40 Hz) z′-coherence values during sleep, wakefulness and 10 min following ketamine administration.

**Animal/Cx**		**AW**	**QW**	**NREM**	**REM**	***K***	***F***
**C1**	**Ppr-Ppl**	1.13 ± 0.03	0.84 ± 0.07	0.77 ± 0.03	0.55 ± 0.03	0.6 ± 0.03^abc^	28
	**S1l-Ppl**	0.98 ± 0.04	0.41 ± 0.03	0.53 ± 0.03	0.33 ± 0.01	0.28 ± 0.01^abc^	87
**C2**	**Pfr-Ppr**	0.90 ± 0.06	0.34 ± 0.05	0.16 ± 0.01	0.10 ± 0.01	0.11 ± 0.01^abc^	78
	**Ppr-Ppl**	0.95 ± 0.04	0.46 ± 0.05	0.27 ± 0.04	0.20 ± 0.03	0.51 ± 0.03^abc^	60
**C3**	**Ppr-Ppl**	0.43 ± 0.01	0.34 ± 0.04	0.05 ± 0.01	0.05 ± 0.01	0.04 ± 0.01^abc^	98
	**S1r-Ppr**	0.97 ± 0.02	0.60 ± 0.04	0.48 ± 0.01	0.48 ± 0.01	0.42 ± 0.03^abcd^	88
**C4**	**M1r-V1r**	0.91 ± 0.02	0.18 ± 0.01	0.15 ± 0.01	0.02 ± 0.01	0.01 ± 0.01^abc^	857
**C5**	**Pfr-Ppr**	0.73 ± 0.04	0.59 ± 0.04	0.42 ± 0.07	0.14 ± 0.06	0.12 ± 0.03^abc^	368
	**S1r-Ppr**	0.91 ± 0.04	0.82 ± 0.06	0.62 ± 0.06	0.42 ± 0.03	0.47 ± 0.03^abc^	28

*The values represent mean ± standard error. The letters a, b, c, and d show the significance (p < 0.05, ANOVA followed by Tamhane tests) compared to ketamine (K); a vs. AW, b vs. QW, c vs. NREM, and d vs. REM sleep. All the analyses have the same degrees of freedom (4 between groups, 55 within groups). Right cortices are shown for C2–C5, while left cortices are exhibited for C1. C1–C5 identification name of the animals. M1, primary motor cortex; Pf, prefrontal cortex; Pp, posterior-parietal cortex; S1, somatosensory cortex; V1, primary visual cortex; Cx, cortex*.

### Dose-Response Curve

**Figure 7:**
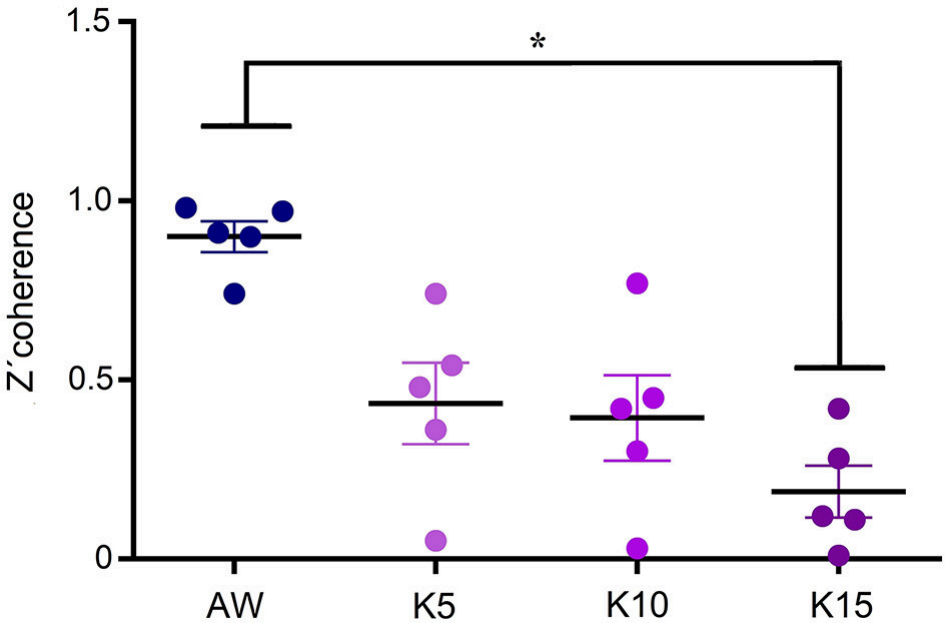
Dose-response curve of gamma z′-coherence. Mean gamma z′-coherence between anterior and posterior cortical areas for the 5 animals during alert wakefulness (AW) and following treatments with 5, 10, and 15 mg/kg (K5, K10, and K15, respectively) of ketamine. Error bars indicate standard error. *F_(3, 19)_ = 40.4, *p* < 0.003.

Figure 7 shows the average gamma z′-coherence values for AW and ketamine at doses of 5, 10, and 15 mg/kg for the 5 animals. In some animals, the doses of 5 and 10 mg/kg had intermediate coherence values between AW and the dose of 15 mg/kg. In others, the doses of 5 and 10 mg/kg had similar gamma z′-coherence than 15 mg/kg.

## Discussion

We have shown that ketamine in sub-anesthetic doses produces a decrease in z′-coherence in the low gamma frequency band (30–45 Hz). This decrease in gamma z′-coherence was similar to that occurring during REM sleep. Furthermore, gamma coherence under ketamine was not affected by novel stimuli, which in basal conditions alert the animal causing a large increase in gamma coherence. On the contrary, 30–45 Hz gamma power remained at a level similar to that observed in QW and REM sleep, but greater than during NREM sleep.

### Technical Considerations

We used the cat as the animal model because it has well-defined, consolidated sleep and waking states. The recordings were obtained in semi-restricted conditions, which has the advantage that differences among states are the states *per se*; postures or movements did not influence the recordings. Furthermore, this condition reduces the possibility of artifacts. This animal model has also the advantage that 30–45 Hz EEG “bursts” of 200–500 ms and ~25 μV, can be observed directly in the raw recordings (38, 48). It is important to highlight that in the cat, low (30–45 Hz) gamma oscillations are highly reactive to alertness and behavioral states (38, 48, 51, 52). On the contrary, high (50–100 Hz) gamma does not respond to stimuli that alert the animals (48, 52). Because of its importance, in the present study we focused in this narrow (30–45 Hz) gamma band. However, we are carrying out new studies analyzing not only on high gamma, but also the high frequency band (110–160 Hz) that is known as high frequency oscillations (HFO). HFO may have a role in psychosis, because experiments in rats have shown that ketamine increases HFO in all depths of the CA1-dentate axis (58), and HFO coherence increases between perceptual cortices of the same hemisphere during REM sleep (59).

In previous studies of our group we discarded the presence of possible artifactual signals that may bias the quantification of the gamma coherence (48, 51). In addition, as it is shown in Supplementary Figure 2, gamma coherence was almost 0 during AW following the subrogation (shuffling) of one channel, while the spectral component of the shuffled channel remained similar to the original one.

From the pharmacological point of view, the maximal dose of ketamine that we used was 15 mg/kg. Hence, it is important to highlight that when it is administered as a single agent, the anesthetic dose of ketamine in cats is ≥25 mg/kg (60–62).

In pilot studies in 3 cats, we administered ketamine (15 mg/kg) and analyzed the behavior in freely-moving conditions. In rodents, NMDA-R antagonists generate a behavioral syndrome characterized by hyperlocomotion, ataxia signs and stereotypies (21, 22). Hyperlocomotion was not observed in our experiments, nor in previous studies in cat (63, 64). Moreover, an increase in motor activity was neither detected in semi-restricted condition. On the contrary, ~5 min following the injection of ketamine, the animals lay down on the floor unable to stand up (i.e., an ataxia-like effect), but responded to sound stimulus directing the gaze toward the sound source. In the absence of stimuli, the cats moved their head from one side to the other (i.e., a head-weavings like behavior, described in rodents, and defined as stereotypies characterized as lateral side-to-side movement of the head without locomotion). The animal also retained muscular tone, showed hyper-salivation and dilated pupils. Three to four hours following ketamine administration, the cats recovered completely.

### REM Sleep as a Natural Model of Psychosis

Hobson (28) considers that dream experiences (the cognitive counterpart of REM sleep) have the following similarities with psychosis (28). I. The intense visual images of dream experiences, which are like the visual hallucinations that frequently occur in toxic states caused by substances that alter the chemistry of the brain (toxic psychosis). II. The conviction that the events of physically impossible dream experiences are real, which is like the delusional belief that is the hallmark of all psychosis. III. The inability to recognize that we are dreaming, which is similar to the tenacity that paranoids cling to false belief. IV. The stories we invent to explain improbable and impossible imaginary events during dreams, which are like the confabulations of Korsakoff's syndrome. Hobson concludes, that “dreaming is, by definition, a psychosis.”

It has been proposed that dreams characteristics, such as the violation of physical laws, inconsistencies in time, space and characters, come from the decrease in dorsolateral prefrontal brain activity that characterizes both psychosis and REM sleep (31, 65–69).

From the electrophysiological point of view, wakefulness in psychotic patients and REM sleep in normal subjects show a similar strong activation of the EEG (30).

Regarding neurochemical aspects, there are two main hypotheses relating psychosis and dreams; these hypotheses involve dopamine and glutamate neurotransmission (30). As abovementioned, NMDA-R antagonists induce psychotic symptoms but at the same time generate vivid (mostly unpleasant) dreams (70). On the other hand, the excess of dopamine in the nucleus accumbens can be considered partially responsible for the positive symptoms of the psychosis (71). In addition, indirect dopamine agonists, such as amphetamine induce both psychotic symptoms and vivid (nightmares) dreams (72, 73). Consistent with this, it was determined that dopamine release in the nucleus accumbens is maximal during REM sleep (74). Antipsychotics drugs, which antagonize the action of dopamine, suppress both psychotic symptoms and dream experiences (75, 76).

Finally, the neurobiological characteristics of REM sleep are candidates endophenotypes of psychosis, and REM sleep is considered a neurobiological model of this mental disorder (28–30).

### Ketamine as a Pharmacological Model of Psychosis

The glutamatergic NMDA-R is expressed ubiquitously in the central nervous system. Clinical and preclinical studies suggest that the NMDA-R is involved in the pathophysiology of the psychotic disorders (77–79). Post-mortem studies of patients with schizophrenia found a decrease in NMDA-R in dorsolateral prefrontal cortex and hippocampus (80). Furthermore, as mentioned in the Introduction, sub-anesthetic doses of NMDA-R antagonists, including ketamine, are widely used to mimic some of the symptoms of psychosis.

### Power and Gamma Coherence During REM Sleep (Natural Model of Psychosis)

Previous studies of our group have shown that the gamma power during REM sleep of the cat is similar to that of QW. On the contrary, gamma coherence is almost absent during REM sleep (45, 48, 51, 52). Similar results have been observed in rats (45, 49, 81), and humans (46, 47). In this regards, the uncoupling of the EEG activity between executive and perceptual regions during REM sleep has been related to the bizarre characteristics of dreams (69).

### Power and Gamma Coherence Under Sub-anesthetic Doses of Ketamine (Pharmacological Model of Psychosis)

Our results demonstrated that sub-anesthetic doses of ketamine reduce gamma coherence between different neocortical areas at a similar level to that of REM sleep, although the gamma power remains comparable to QW.

In agreement with our results, Pinault (82) demonstrated in rats a dose-dependent increase in fronto-parietal gamma power that occurs even at relatively low doses of ketamine (2.5 mg/kg) and MK801 (0.06 mg/kg, the most potent NMDA-R antagonist) (82). Other authors obtained similar results (17, 83–86).

With respect to gamma coherence, and also in harmony with our results, Pal et al. (87) identified in the rat a decrease in coherence with an anesthetic (150 mg/kg) dose of ketamine. They demonstrated that ketamine-induced unconsciousness was associated with reduction of power in high gamma bandwidths (>65 Hz), and in coherence in the whole gamma range. This fact was accompanied by a significant increase in acetylcholine (ACh) concentrations in the prefrontal cortex. Compared with the unconscious state, recovery of righting reflex was marked by a further increase in ACh concentrations, increases in low gamma band (25–55 Hz) power, and an increment in power and coherence in high gamma frequencies (>65 Hz). On the contrary, Akeju et al. (88) found an increase in gamma power and coherence in the anesthetic induction with ketamine in humans, probably because the coherence analysis was performed in standard EEG recordings (scalp electrodes) between frontal electrodes located at a short distance.

Ketamine seems to block NMDA-Rs (that are excitatory) in GABAergic cortical interneurons more efficiently than in pyramidal neurons. This reduction in the excitatory inputs onto the interneurons would lead to a decrease in the release of GABA at the synapses between interneurons and pyramidal neurons (89, 90), and a disinhibition of pyramidal neurons (91). This may explain why ketamine is associated with a greater use of cerebral glucose and blood flow (92, 93), and with an increase in gamma power (see above). However, it is probable that coupling between different cortical areas would be mediated by glutamate acting through NMDA-R. So, when these receptors are blocked by ketamine, the coupling between different distant cortical areas decreases, but without decreasing local synchronization.

Ketamine induces psychotic symptoms during the first 40–60 min after its administration (94). The temporal dynamics of these symptoms coincides with the dynamic evolution of the decrease in gamma coherence observed in our experiments. This lack of coupling of gamma frequency activity during the maximum effect of ketamine could be involved in the peculiarities of cognitive operations that occur under the effect of ketamine.

Do patients suffering of psychotic disorders present dysfunctions in the EEG gamma activity? Altered gamma oscillations have been observed in psychosis (95–97). In fact, schizophrenia has dysfunction of GABAergic cortical interneurons that express the calcium-binding protein parvalbumin, particularly in the prefrontal cortex (98, 99). These neurons have been involved in the generation of gamma oscillations (100).

White and Siegel (101) showed that in psychosis, gamma power is increased during QW (101). Moreover, during sleep there is a decrease in beta and gamma coherence between the right frontal and central right areas (102). In addition, under visual or auditory sensory stimulation there is an increase in gamma activity (30–50 Hz), while the phase coherence is reduced in psychotic patients (103–108). These findings agree with lack of increment in gamma coherence in response to sensory stimuli following the administration of ketamine (Figures 2, 3).

## Conclusions

Functional interactions between cortical areas in the gamma frequency band decrease in a similar way in both experimental models of psychosis: ketamine and REM sleep. This decoupling of gamma frequency activity may be involved in the cognitive characteristics shared by dream experiences and psychosis.

## Data Availability Statement

The data that supported the findings of this study are available from the corresponding author upon reasonable request.

## Author Contributions

PT provided the financial support. PT and SC-Z performed the experimental design. SC-Z, MC, JG, and PT performed the experimental procedures and were involved in the discussion and interpretation of the data. SC-Z, MC, and JG analyzed the data. SC-Z, MC, and PT wrote the manuscript. CS, AN, and SM critically revised the manuscript and added important intellectual content to it. All the authors reviewed and approved the definitive version of the manuscript.

### Conflict of Interest Statement

The authors declare that the research was conducted in the absence of any commercial or financial relationships that could be construed as a potential conflict of interest.
