# Supplementary figures and images for: EEG 40 Hz Coherence Decreases in REM Sleep and Ketamine Model of Psychosis
Source: Front Psychiatry. 2019 Jan 17;9:766. doi: 10.3389/fpsyt.2018.00766 (PMC6345101; doi:10.3389/fpsyt.2018.00766)

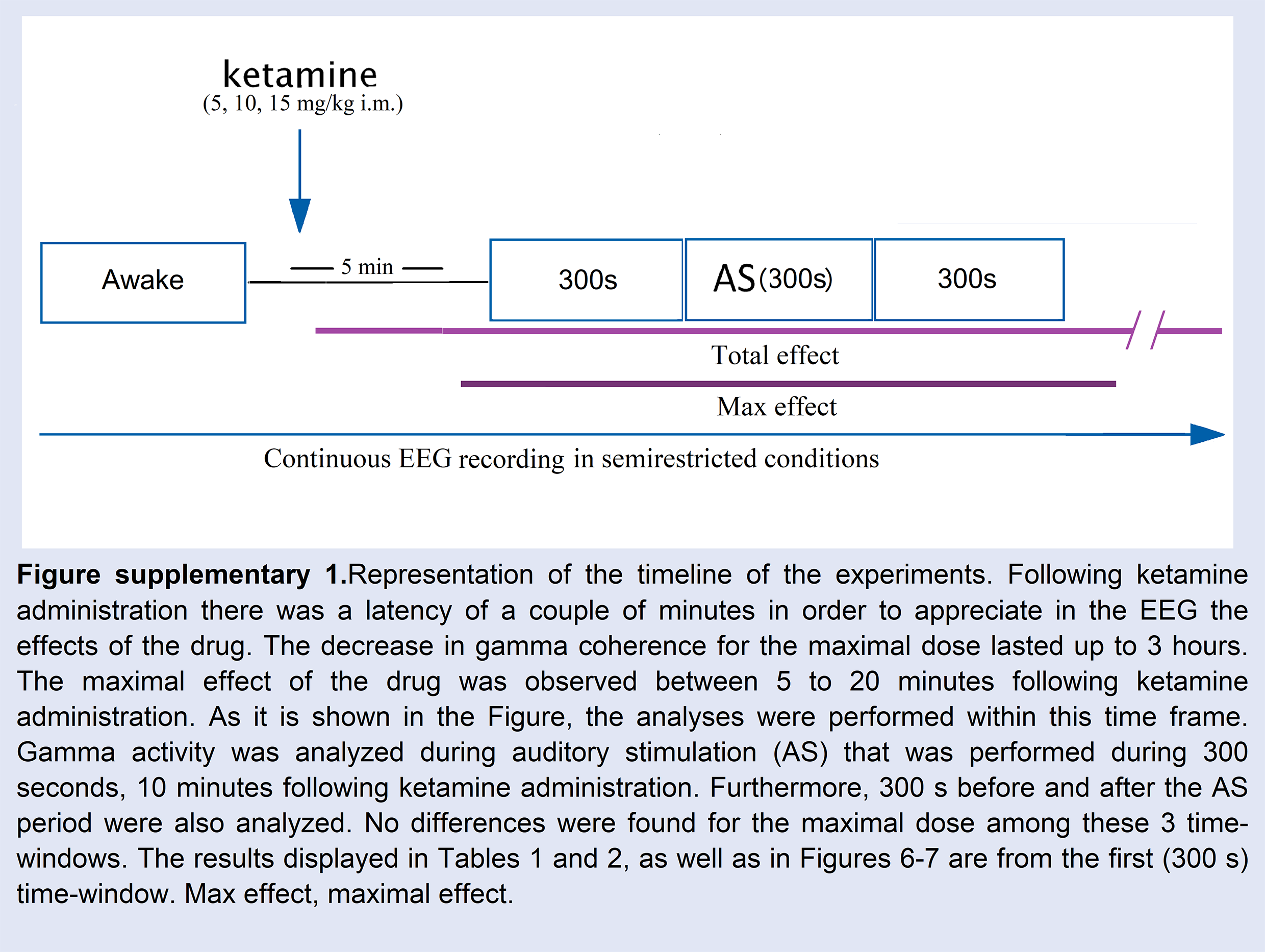

Supplement: Supplementary file 1 [file Image_1.TIF]

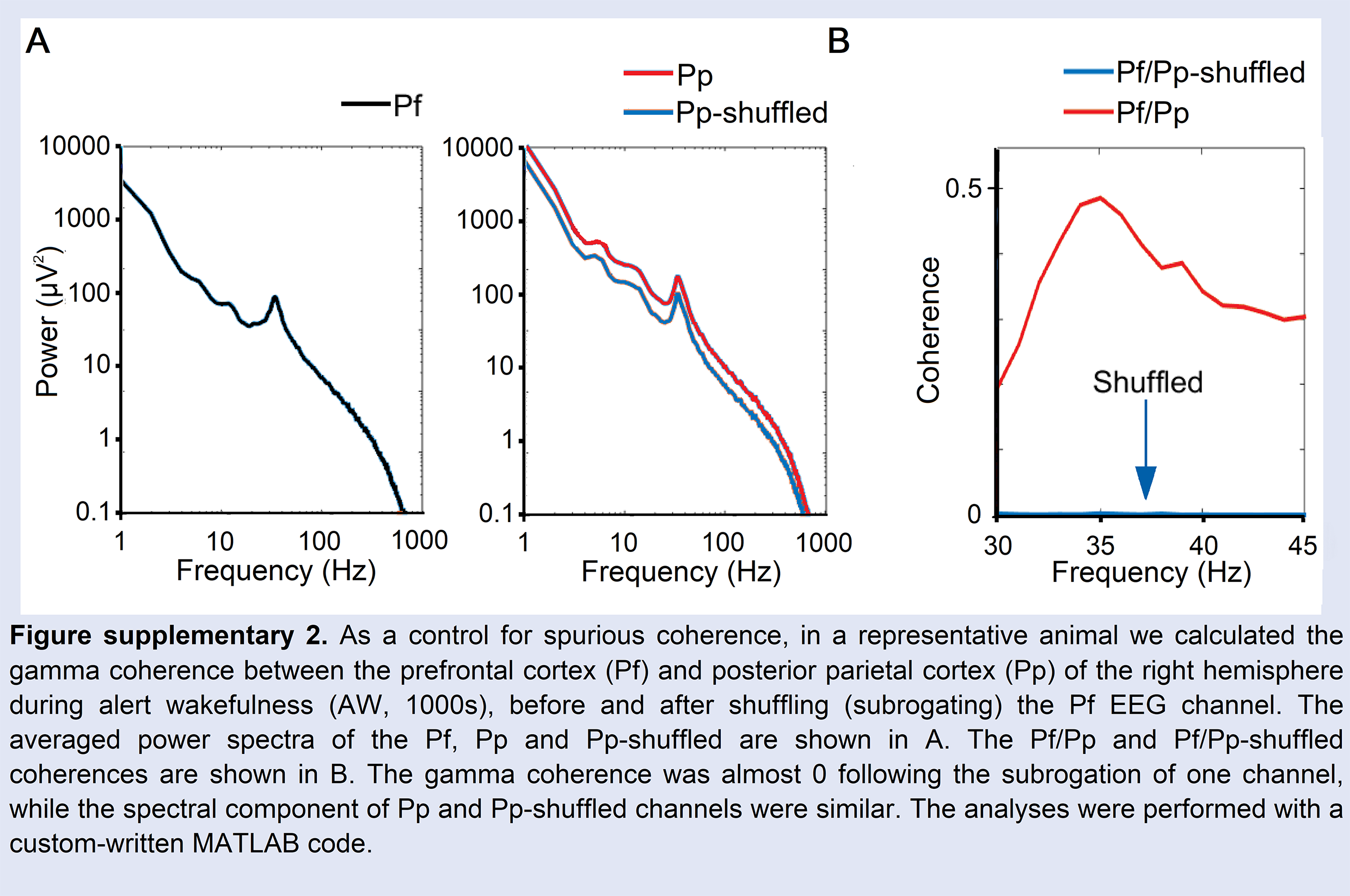

Supplement: Supplementary file 2 [file Image_2.TIF]
